# Circadian rhythm of activin A and related parameters of mineral metabolism in normal and uremic rats

**DOI:** 10.1007/s00424-019-02291-2

**Published:** 2019-06-24

**Authors:** Anders Nordholm, Søren Egstrand, Eva Gravesen, Maria L. Mace, Marya Morevati, Klaus Olgaard, Ewa Lewin

**Affiliations:** 1 Nephrological Department, Herlev Hospital, University of Copenhagen, 2730 Herlev, Denmark; 2 Nephrological Department, Rigshospitalet, University of Copenhagen, 2100 Copenhagen, Denmark

**Keywords:** Activin A, Circadian rhythm, FGF23, Klotho, Phosphate, CKD

## Abstract

Activin A is a new fascinating player in chronic kidney disease-mineral and bone disorder (CKD-MBD), which is implicated in progressive renal disease, vascular calcification, and osteodystrophy. Plasma activin A rises early in the progression of renal disease. Disruption of circadian rhythms is related to increased risk of several diseases and circadian rhythms are observed in mineral homeostasis, bone parameters, and plasma levels of phosphate and PTH. Therefore, we examined the circadian rhythm of activin A and CKD-MBD-related parameters (phosphate, PTH, FGF23, and klotho) in healthy controls and CKD rats (5/6 nephrectomy) on high-, standard- and low-dietary phosphate contents as well as during fasting conditions. Plasma activin A exhibited circadian rhythmicity in healthy control rats with fourfold higher values at acrophase compared with nadir. The rhythm was obliterated in CKD. Activin A was higher in CKD rats compared with controls when measured at daytime but not significantly when measured at evening/nighttime, stressing the importance of time-specific reference intervals when interpreting plasma values. Plasma phosphate, PTH, and FGF23 all showed circadian rhythms in control rats, which were abolished or disrupted in CKD. Plasma klotho did not show circadian rhythm. Thus, the present investigation shows, for the first time, circadian rhythm of plasma activin A. The rhythmicity is severely disturbed by CKD and is associated with disturbed rhythms of phosphate and phosphate-regulating hormones PTH and FGF23, indicating that disturbed circadian rhythmicity is an important feature of CKD-MBD.

**Electronic supplementary material:**

The online version of this article (10.1007/s00424-019-02291-2) contains supplementary material, which is available to authorized users.

## Introduction

Activin **A** is a member of the transforming growth factor beta (TGF-β) family of proteins produced by many cell types throughout development [7]. It participates in regulation of several biological processes, including cell differentiation, proliferation, and inflammatory response. Systemic activin A levels are increased in postmenopausal women, aging, and patients with type 2 diabetes mellitus [2, 23, 45, 77]. Recently, the first report on systemic activin A elevation in humans with uremia was provided. It was found that in patients with chronic kidney disease (CKD), serum activin A levels increased early in the progression of renal insufficiency [43]. Activin A not only has an important role in kidney development and repair [50], but also an essential role in kidney diseases, such as acute renal failure or progressive renal fibrosis [1, 50, 80]. It was observed that renal expression of activin A was induced in kidney injury stressing the concept that an endocrine factor, which is produced in kidney failure, disrupts organ homeostasis outside the kidney and that activin A might be such a circulating factor [58].

Activin A mediates its biological effects through a complex of transmembrane receptor serine/threonine kinases. Activin A binds to activin A receptor type II (ActRllA), then forms a complex with ALK4. Phosphorylation of ALK4 activates Smad2/3 and forms a complex together with Smad4 that translocate to the nucleus to regulate gene expression [7]. Preventing ActRllA signaling with a fusion protein, ACE-011, which contains the ActRIIA domain derived from the human receptor resulted in elevation of osteoblastic bone formation markers and reduction of osteoclastic bone resorption markers in healthy postmenopausal women [64]. Recently, systemic activation of activin receptors in the kidney, skeleton, vasculature, and heart in CKD mouse models of diabetic nephropathy and Alport syndrome has been reported [1, 73, 80]. Moreover, the treatment with activin A-binding protein, follistatin, or with RAP-011, a ligand trap of ActRllA, has revealed an amelioration of renal fibrosis and chronic kidney disease-mineral bone disorder (CKD-MBD) findings in CKD models [1, 33, 51, 73, 80]. As such, increased systemic activin A can be seen as a biomarker of CKD-MBD that can be targeted for CKD-MBD prevention and therapy.

CKD-MBD is a major cause of excess mortality associated with CKD [55]. CKD-MBD begins early in the course of kidney disease and consists of renal osteodystrophy, vascular calcification (VC), and cardiac disease together with elevations of plasma phosphate (P) and fibroblast growth factor 23 (FGF23) as well as decrease of klotho [46, 47, 55]. FGF23 is an important hormone secreted from osteocytes that regulates PTH and vitamin D metabolism and augments renal P excretion [49, 69, 76]. The phosphaturic action of FGF23 requires klotho, an antiaging protein which functions as co-receptor for signal transduction [38, 78]. In more advanced CKD-MBD secondary hyperparathyroidism (sHPT), calcitriol deficiency, and hyperphosphatemia develop [31, 40, 46]. Elevated plasma P levels are associated with several deleterious endpoints in CKD patients including sHPT, arterial hypertension, extra-skeletal calcifications, cardiovascular disease, fracture rates, and all-cause mortality [13, 34, 55, 63]. Even in individuals with normal kidney function, plasma P levels are associated with long-term development of VC [16]. The possible mechanism by which P influences cardiovascular mortality is by the involvement of extracellular P in promoting the expression of an osteogenic phenotype in vascular myocytes [65, 68]. A decrease in renal klotho expression is a new component of CKD-MBD as systemic klotho is derived from the kidney [37, 44]. The loss of klotho in CKD is associated with VC, cardiac hypertrophy, and osteodystrophy [27, 29, 81]. As such, CKD has similarity to the phenotype of klotho hypomorph mice characterized by accelerating aging [28]. Replacement of klotho has been shown to be efficacious in both conditions [28] and klotho is regarded a vasculo-protective factor [42]. In time, VC becomes manifest and irreversible even though improving hyperphosphatemia and gene expression profile can be reached with various pharmacological manipulations [21, 22]. However, inhibition of ActRllA signaling, in early CKD mouse models, improves VC and renal fibrosis and increases renal klotho [1]. As such, increased systemic activin A and activated systemic ActRllA signaling may represent a new critical component of CKD-MBD, which is implicated in the onset and progression of the disease. Furthermore, activin A may be implicated not only in CKD-MBD, but also in premature aging in CKD, as some manifestations of the phenotypes of CKD-MBD overlap with that of premature aging, such as decrease in klotho, medial VC, and osteoporosis [41].

Our hypothesis is that an increase in circulating levels of activin A in CKD is associated with a disruption of the circadian rhythm (CR) of plasma activin A.

Proper rhythms in hormone secretion, metabolism, cell cycle, and behavior are maintained by a circadian clock; an endogenous, self-sustaining pacemaker that operates with a periodicity of 24 h [24]. Disruption in the proper circadian clock results in detrimental effects on the mammalian physiology [52]. Circadian rhythmicity is observed in mineral homeostasis and bone parameters and has been shown for plasma P and parathyroid hormone (PTH) [32, 53, 62, 70, 74]. Bone is the main reservoir of calcium and P. Activin A seems to be a positive regulator for osteoclastic development and bone resorption and a negative regulator for osteoblastic bone formation in vivo [72]. The mammalian CR field has historically focused on the suprachiasmatic nucleus in the hypothalamus, which is essential for directing cycles of locomotor activity [24]. However, in addition to this central pacemaker, a molecular clock has been found in several peripheral tissues such as intestine, vasculature, adipose tissue, and kidney [52, 71]. For most tissues, it is still needed to establish which specific input determines the phase of the local cellular clock.

The CR of activin A has not previously been examined. In the present investigation, the CR of plasma activin A is studied in normal and long-term CKD rats together with 24-h rhythms of P, calcium, PTH, and the new P-regulating hormones FGF23 and klotho. As plasma P and PTH levels may be entrained by nutrient availability, we examined how these rhythms are influenced by depletion of dietary P or high P content in the diet, and by fasting.

The results of the present investigation in the rat established for the first time the existence of the CR of circulating activin A. This rhythmicity is disturbed in CKD rats and is associated with disturbed CRs of P and the P-regulating hormones PTH and FGF23.

## Methods

### Animals

Male Wistar rats (Charles River, Germany) were housed in a temperature-controlled environment with a 12-h light-dark cycle (light 07:00–19:00 h). They had ad libitum access to water and food. The study was approved by the Danish Animal Inspectorate (Reference no. 2012-DY-2934-00023 and 2017-15-0201-01214) and executed in accordance with national guidelines for use of laboratory animals.

### Design

Adult rats were acclimatized for 1 week before randomization to CKD or control. Under anesthesia with Hypnorm/Midazolam (Panum Institute, Denmark), CKD was introduced by one-step 5/6 partial nephrectomy (PNX). Control rats received a standard-phosphate (SP) diet (0.9%Ca, 0.7%P, 600 IU vitamin D3 per kg food). CKD rats received high-phosphate (HP) diet (0.9%Ca, 1.4%P, 600 IU vitamin D3 per kg food), standard-phosphate (SP) diet, or low-phosphate (LP) diet (0.9%Ca, 0.2%P, 600 IU vitamin D3 per kg food), (Altromin Spezialfutter, Germany). The duration of CKD was 24 weeks.

The evaluation of circadian rhythms (CR) was followed in four groups of rats: PNX HP (*N* = 26), PNX SP (*N* = 8), PNX LP (*N* = 8), and controls (*N* = 26). Blood samples were collected at times 08:00, 14:00, 20:00, and 02:00 h according to a prearranged scheme ensuring no difference in the order of phlebotomy within and between groups. All rats were only phlebotomized once daily. Two weeks later, the fasting experiment was performed in the same four groups of rats. Blood samples were collected in the morning on two consecutive days. Non-fasting samples were collected on day one and fasting samples on day two. Diet was removed around 16:00 on day one resulting in 16 h of fasting.

### Biochemistry

Tail blood were drawn and analyzed immediately by ABL 505 (Radiometer, Denmark) for ionized calcium, sodium, potassium, and pH. One milliliter of blood was drawn into heparinized tubes and immediately centrifuged. Plasma was separated, divided into several tubes (to avoid freeze-thaw cycles), and stored at − 80 °C until analysis. Blood urea nitrogen (BUN), phosphate (P), and total calcium were measured at the Department of Clinical Biochemistry, Rigshospitalet, Denmark. Activin A was measured by the Quantikine ELISA rat activin A immunoassay (R&D Systems, USA) with intra- and inter-assay variations of 4% and 5%, respectively. PTH and FGF23 were measured by the rat bioactive PTH ELISA assay (Immutopics, USA) and the intact FGF23 ELISA assay (Kainos Laboratories, Japan), respectively. In our lab, the intra- and inter-assay variations were 4% and 9% in the PTH assay [30], and 2.5% and 5% in the FGF23 assay [20]. Klotho was kindly measured by an immunoblot-immunoprecipitation assay at the George M. O’Brien Kidney Research Core Center (Uni. Texas Southwestern, USA) [4].

### Statistics

All analyses were performed using GraphPad Prism 7.02 and RStudio 1.0.153 (cosinor and cosinor2 packages). *p* ≤ 0.05 was considered significant.

Circadian fluctuations are presented as graphs with mean ± SEM. Difference between means within the same group was calculated by repeated measures one-way ANOVA with Tukey’s multiple comparison test. Between-group analyses of circadian fluctuations were performed by two-way ANOVA with Bonferroni’s multiple comparison test. Circadian rhythmicity was confirmed and presented by cosinor analysis. For cosinor analyses, data was fitted to a linear model using the least squared method minimizing the residual sum of the squares:

$$ Y(t)= Mesor+\beta \cdotp \cos \frac{2\pi \cdotp t}{24}-\gamma \cdotp \sin \frac{2\pi \cdotp t}{24}+e(t) $$

where *t* = zeitgebertime (zt), β = A ∙ cos φ, *γ* =  − A ∙ sin φ, *e*(*t*) is the error term, A = amplitude, φ = acrophase. The period is fixed at 24 h. Significant rhythm is found when the 95% confidence intervals of the acrophase do not include Mesor (Midline Estimation Statistic of Rhythm) [11]. Acrophase is rounded to nearest whole hour. The R software package “cosinor” was used for fitting to a cosinor model—presented as a double-plot (i.e., 48 h). The R software package “cosinor2” was used for evaluating the power of the cosinor models using an *F* test and coefficient of determination (*r*^2^).

Paired Student’s *t* test or Wilcoxon matched-pairs signed rank test was used for comparing non-fasting and fasting within the same group. Parameters between groups were calculated with unpaired Student’s *t* test or Mann-Whitney *U* test.

## Results

### Circadian rhythm of plasma activin A

**Fig. 1:**
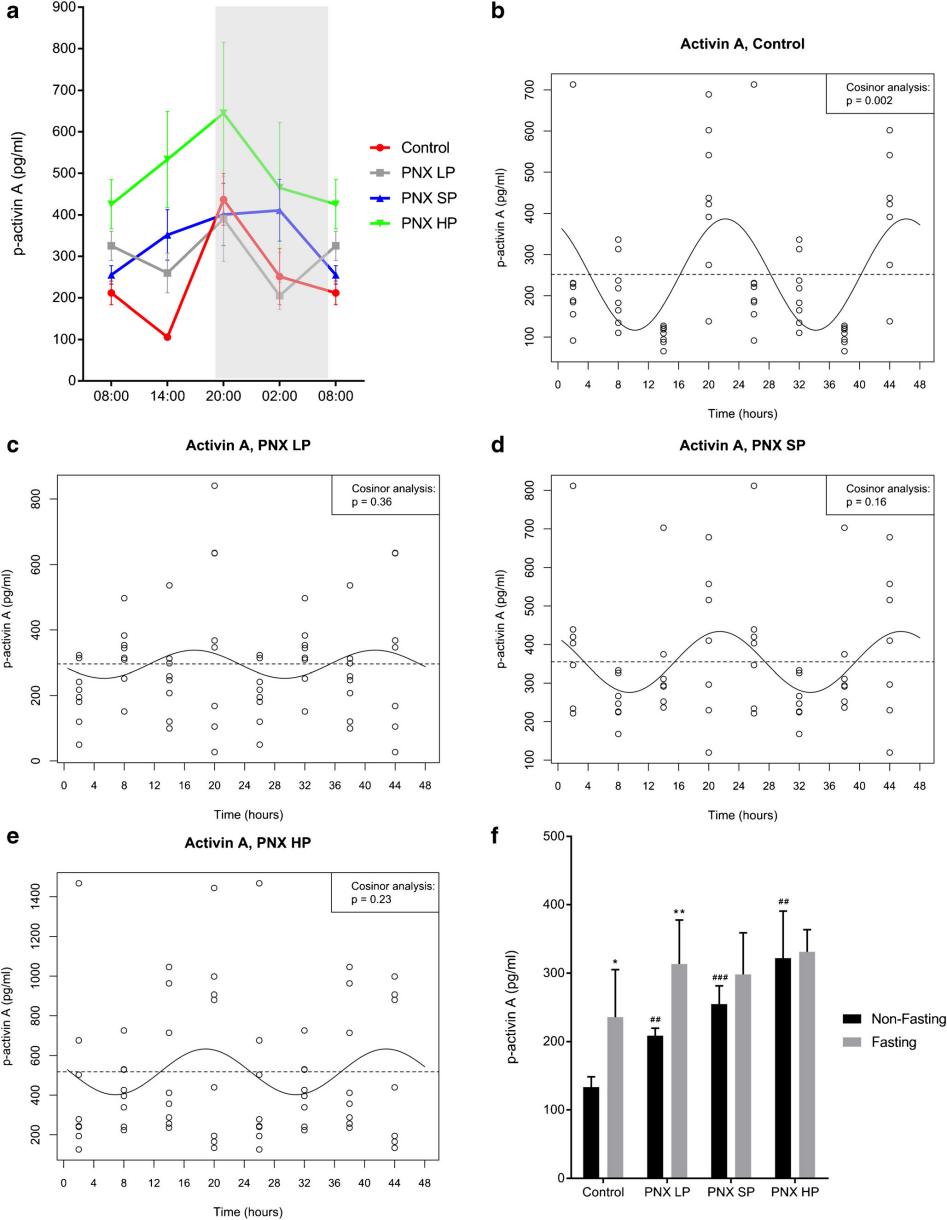
Plasma activin A exhibits circadian rhythmicity in healthy control rats, but the rhythm is obliterated by CKD. **a** Circadian rhythm of plasma activin A in healthy controls (red), PNX LP (gray), PNX SP (blue), and PNX HP rats (green). Controls showed circadian rhythm (*p* < 0.001) with a fourfold higher value at 20:00 compared with 14:00. The rhythm was obliterated in all PNX groups. **b**–**e** Cosinor analysis confirmed the existence of circadian rhythm in healthy controls, *p* < 0.01, with acrophase at 22:00 (**b**) and obliteration of rhythm in PNX LP (**c**), PNX SP (**d**), and PNX HP rats (**e**). **f** Non-fasting (black) and fasting (gray) plasma activin A levels in controls, PNX LP, PNX SP, and PNX HP rats. Fasting caused an increase in controls and PNX LP rats, but not in PNX SP or PNX HP rats. All PNX groups had higher non-fasting activin A levels as compared with controls. *p < 0.05 and **p < 0.01 (compared with non-fasting). ^##^*p* < 0.01 and ^###^*p* < 0.001 (compared with non-fasting controls). PNX 5/6 partial nephrectomy, LP low-phosphate diet, SP standard-phosphate diet, HP high-phosphate diet. Mean ± SEM (**a**, **f**). Cosinor fit (**b**–**e**).

Plasma activin A showed CR in normal rats (ANOVA *p* < 0.001) with a fourfold higher value at 20:00 compared with 14:00, *p* < 0.01 (Fig. 1a). The significant diurnal 24-h rhythm of plasma activin A in normal rats was confirmed by cosinor analysis (*p* < 0.01), showing acrophase at 22:00 (Fig. 1b). This circadian rhythmicity was obliterated in all CKD rats (cosinor analysis PNX LP *p* = 0.36, PNX SP *p* = 0.16, and PNX HP *p* = 0.23), even though some fluctuations of plasma activin A levels were seen in the PNX rats on different dietary P content (Fig. 1a, c–e).

The CR of activin A in normal rats makes the time of sampling decisive for detection of differences in plasma levels between normal and CKD rats as all PNX groups had higher activin A levels when directly compared with controls at 08:00 and 14:00 (*p* < 0.05) but not at 20:00 and 02:00 (Fig. 1a). The only exception was PNX SP, which did not differ from controls at 08:00.

**Fig. 2:**
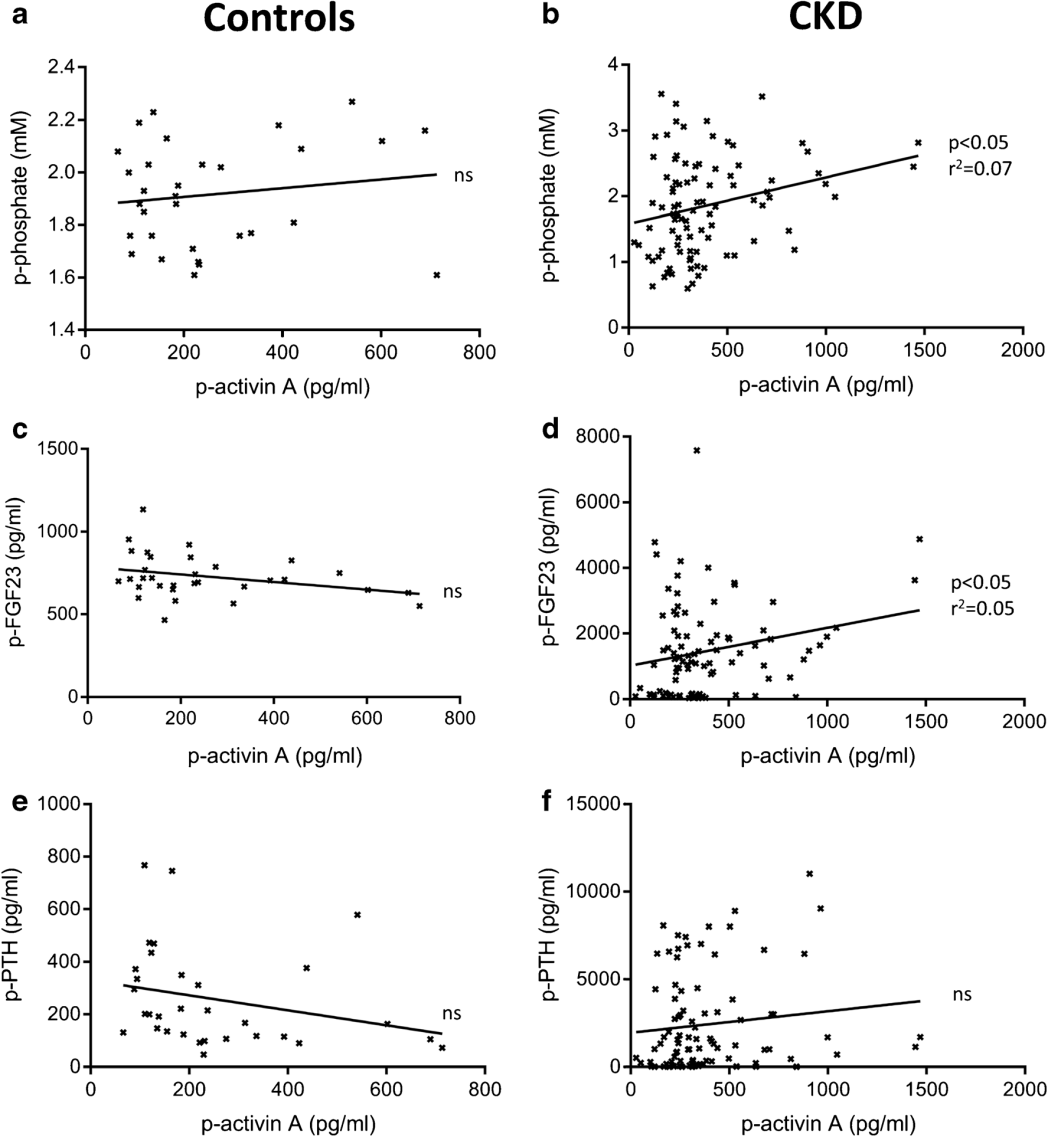
Plasma activin A correlated with plasma phosphate and FGF23 in CKD rats but correlations were absent in controls. **a**, **b** Correlation between activin A and phosphate in controls (**a**) and CKD rats (**b**) revealed a positive correlation in CKD but not in control rats. **c**, **d** Similarly, a correlation between activin A and FGF23 was absent in controls (**c**) but present in CKD rats (**d**). **e**, **f** No correlation could be demonstrated between activin A and PTH in either controls (**e**) or CKD rats (**f**)

In healthy control rats, plasma levels of activin A did not correlate to plasma P, PTH, or FGF23 (Fig. 2a, c, e). However, in CKD rats, significant correlations appeared between activin A and P (*p* < 0.05, *r*^2^ = 0.07) and activin A and FGF23 (*p* < 0.05, *r*^2^ = 0.05) but not between activin A and PTH (Fig. 2b, d, f). This may indicate different regulations and different sources of circulating activin A in normal and CKD rats.

Rhythm characteristics of cosinor analysis are presented in Supplementary Table 1.

### Circadian rhythm of plasma FGF23, PTH, phosphate, and klotho

**Fig. 3:**
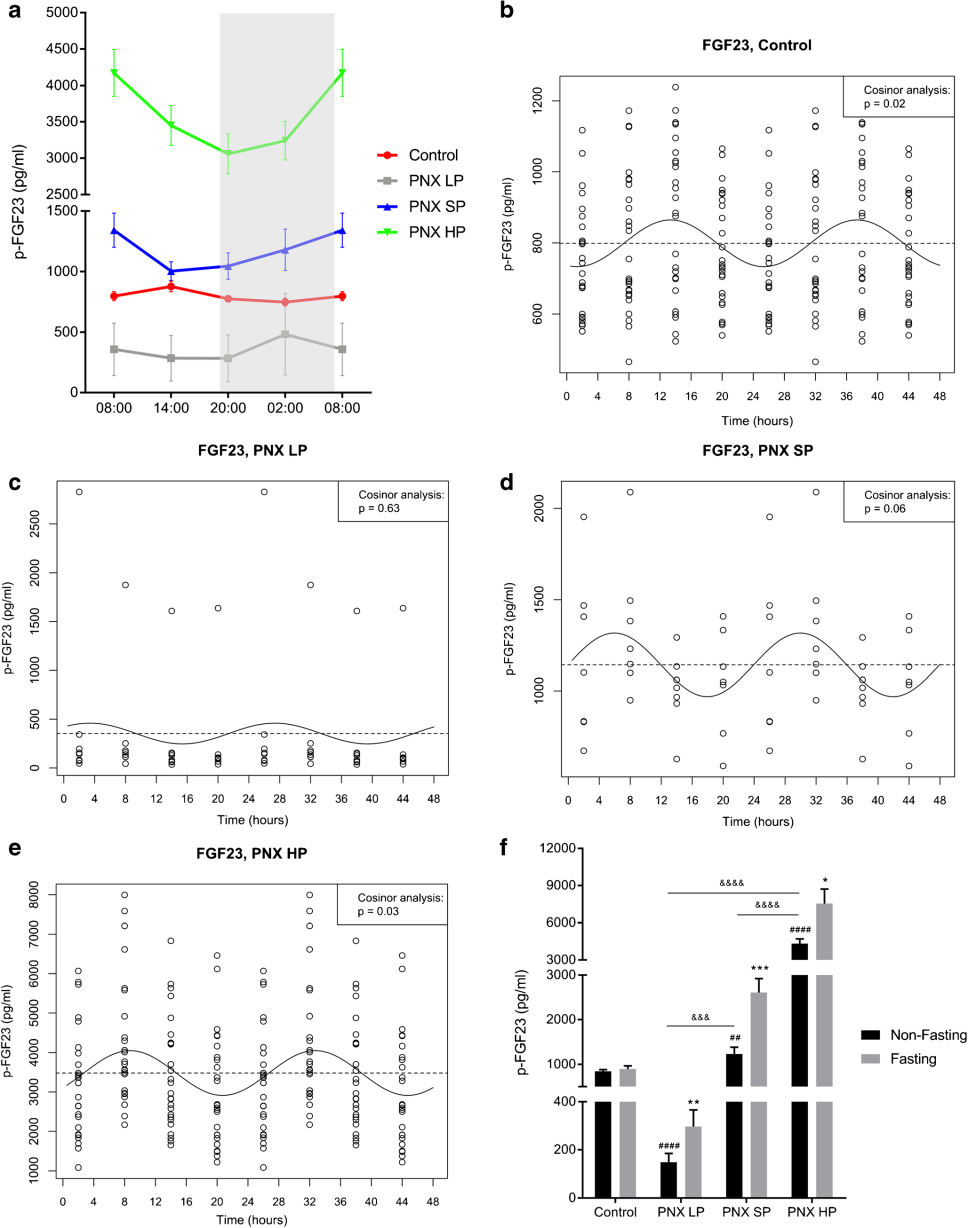
Plasma FGF23 exhibits circadian rhythm in healthy control rats, which is abolished or disturbed in CKD. **a** Circadian rhythm of plasma FGF23 in healthy controls (red), PNX LP (gray), PNX SP (blue), and PNX HP rats (green). Control rats showed significant circadian rhythm (*p* < 0.001). The rhythm was preserved (*p* < 0.0001) but severely disturbed in PNX HP rats with shift of acrophase. In both PNX LP and SP rats, the rhythm was abolished. **b**–**e** Circadian rhythm examined by cosinor analysis confirmed the findings of rhythmicity in healthy controls, *p* < 0.01, (**b**), and PNX HP rats, *p* < 0.05 (**e**) as well as obliteration in PNX LP (**c**) and PNX SP rats (**d**). Acrophase was shifted to 09:00 in the PNX HP group (**e**) compared with 13:00 in controls (**b**). **f** Non-fasting (black) and fasting (gray) plasma FGF23 levels in controls, PNX LP, PNX SP, and PNX HP rats. Fasting caused an increase in plasma FGF23 in all PNX groups but not in controls. *p < 0.05, **p < 0.01, and ***p < 0.001 (compared with non-fasting). ^##^*p* < 0.01 and ^####^*p* < 0.0001 (compared with non-fasting controls). ^&&&^*p* < 0.001 and ^&&&&^*p* < 0.0001. PNX 5/6 partial nephrectomy, LP low-phosphate diet, SP standard-phosphate diet, HP high-phosphate diet. Mean ± SEM (**a**, **f**). Cosinor fit (**b**–**e**).

In control rats, plasma FGF23 had relatively modest but significant alterations during the day (ANOVA *p* < 0.001), (Fig. 3a). Cosinor analysis verified a significant diurnal 24-h rhythm of plasma FGF23 (*p* < 0.05), with acrophase at 13:00 (Fig. 3b).

The P content in the diet had a clear impact on the basal levels of plasma FGF23 in CKD rats with higher concentrations in PNX HP rats (*p* < 0.0001) and lower in PNX LP rats (*p* < 0.01) as compared with control rats at all time points (Fig. 3a), except PNX LP vs control at 02:00—corresponding to the lowest value measured in control rats. Also, in PNX SP rats, the FGF23 levels were higher than in both control and PNX LP rats (*p* < 0.01), except when compared with controls at 14:00—corresponding to the highest value in control rats. The circadian rhythmicity of plasma FGF23 was abolished or disturbed in CKD rats (Fig. 3a, c–e). As such, the CR of FGF23 was obliterated in PNX LP and PNX SP (cosinor analysis *p* = 0.63 and *p* = 0.059) whereas the CR was maintained in PNX HP rats (cosinor analysis *p* < 0.05), but with acrophase shifted from 13:00 in controls to 09:00 in PNX HP rats.

**Fig. 4:**
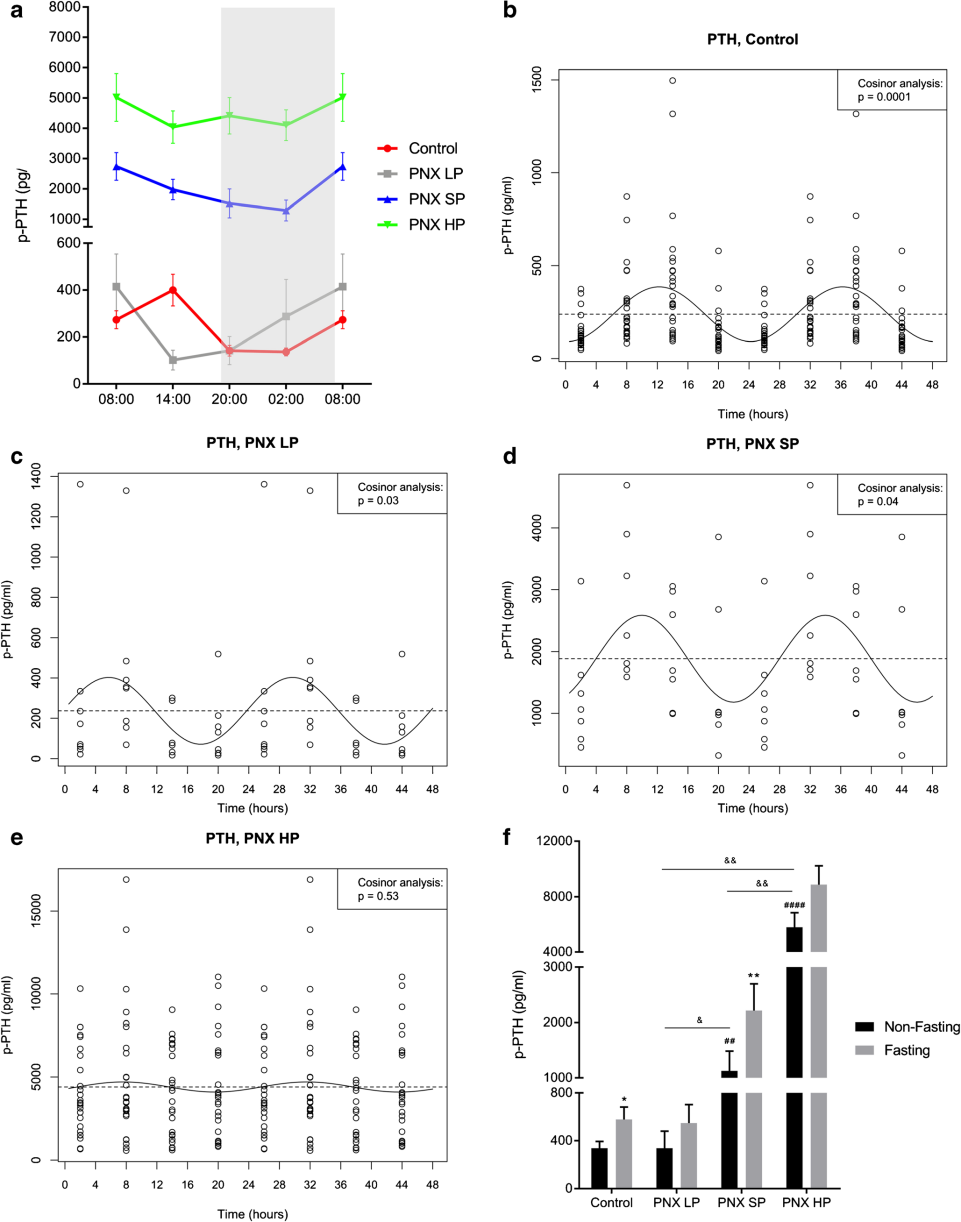
The circadian rhythm of plasma PTH in healthy control rats is obliterated or disturbed in CKD. **a** Circadian rhythm of plasma PTH in healthy controls (red), PNX LP (gray), PNX SP (blue), and PNX HP rats (green). Control rats exhibited circadian rhythm (*p* < 0.001). The rhythm was obliterated or disturbed in CKD rats with shift of acrophase in all PNX groups. **b**–**e** Circadian rhythm examined by cosinor analysis confirmed the rhythmicity in healthy controls, *p* < 0.0001, with acrophase at 12:00 (**b**), and revealed a significant circadian rhythm in both PNX LP, *p* < 0.05 (**c**) and PNX SP rats, *p* < 0.05 (**d**) but the rhythm was completely abolished in PNX HP rats. Both PNX LP and SP rats had disturbed rhythm with shift in acrophase to 06:00 (**c**) and 10:00 (**d**), respectively. **f** Non-fasting (black) and fasting (gray) plasma PTH levels in controls, PNX LP, PNX SP, and PNX HP rats. Fasting caused an increase in plasma PTH in controls and PNX SP rats. *p < 0.05 and **p < 0.01 (compared with non-fasting). ^##^*p* < 0.01 and ^####^*p* < 0.0001 (compared with non-fasting controls). ^&^*p* < 0.05 and ^&&^*p* < 0.01. PNX 5/6 partial nephrectomy, LP low-phosphate diet, SP standard-phosphate diet, HP high-phosphate diet. sHPT secondary hyperparathyroidism. Mean ± SEM (**a**, **f**). Cosinor fit (**b**–**e**)

Control rats had CR of plasma PTH (ANOVA *p* < 0.001), (Fig. 4a). The CR was confirmed by cosinor analysis (*p* < 0.0001), revealing acrophase at 12:00 (Fig. 4b). Again, the circadian rhythmicity was abolished or disturbed in CKD rats (Fig. 4a, c–e). In PNX LP rats, the CR was verified by cosinor analysis (*p* < 0.05) and the development of sHPT was prevented. However, the rhythm was severely disturbed with an earlier peak at 06:00 corresponding to 12:00 in controls. PNX SP rats had significant sHPT (*p* < 0.0001) and significant CR confirmed by cosinor analysis (*p* < 0.05) but the rhythm was disturbed with shift in acrophase to 10:00. The PNX HP group had severe sHPT (*p* < 0.0001) but the CR was completely abolished (*p* = 0.53).

**Fig. 5:**
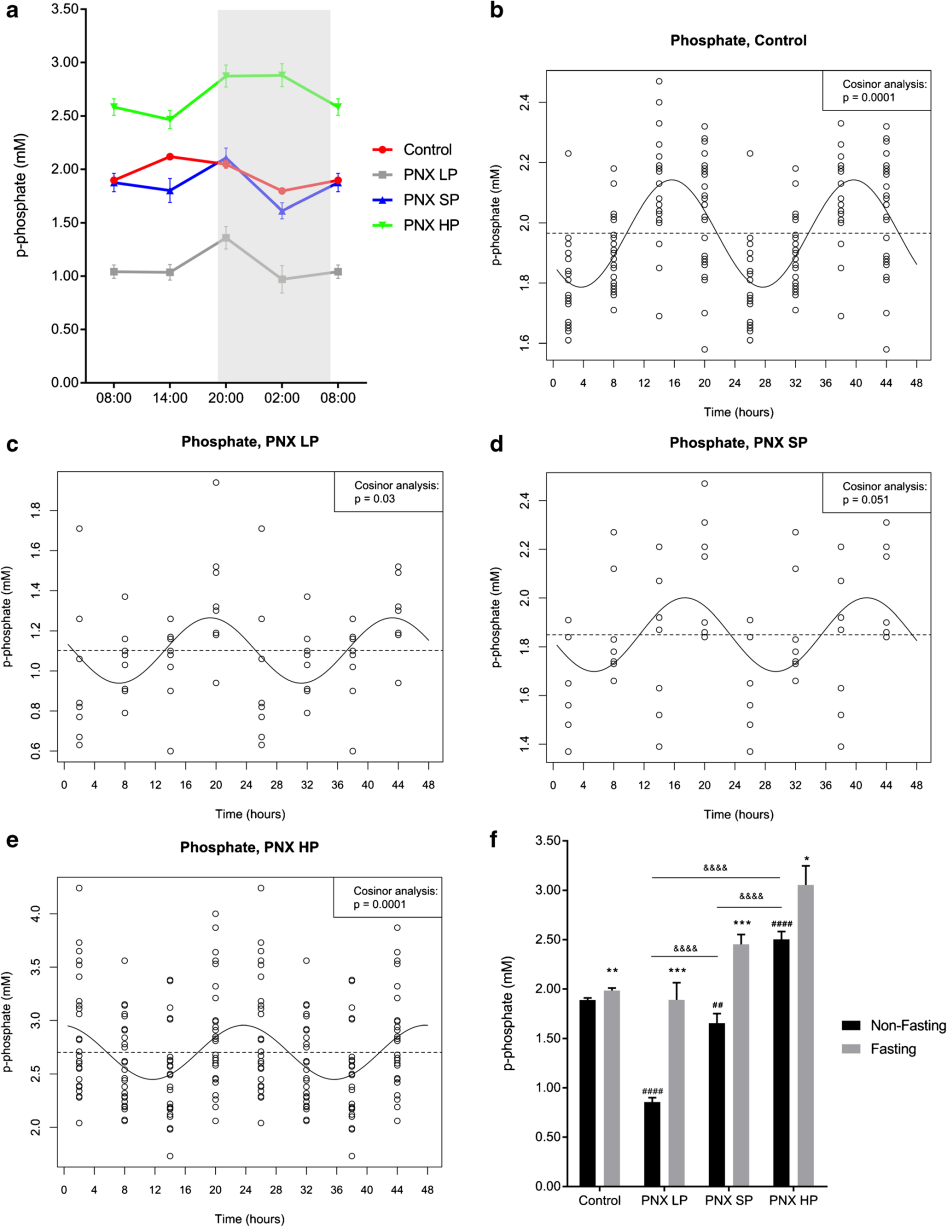
The circadian rhythm of plasma phosphate in healthy control rats is disturbed in CKD. **a** Circadian rhythm of plasma phosphate in healthy controls (red), PNX LP (gray), PNX SP (blue), and PNX HP rats (green). All groups showed significant circadian rhythm (control *p* < 0.0001, PNX LP *p* < 0.05, PNX SP *p* < 0.05, PNX HP *p* < 0.0001). The rhythm was clearly disturbed in CKD rats with peaks at 20:00 in all PNX group, compared with 14:00 in controls. **b**–**e** Circadian rhythm by cosinor analysis confirmed the rhythmicity in healthy controls, *p* < 0.001 (**b**), PNX LP, *p* < 0.05 (**c**), PNX SP, *p* = 0.05 (**d**), and PNX HP rats, *p* < 0.0001 (**e**). All PNX groups had a shift in acrophase from 16:00 in controls (**b**) to 19:00 in PNX LP (**c**), 17:00 in PNX SP (**d**), and 00:00 in PNX HP (**e**). **f** Non-fasting (black) and fasting (gray) plasma phosphate levels in controls, PNX LP, PNX SP, and PNX HP rats. Fasting caused an increase in plasma phosphate in all groups. *p < 0.05, **p < 0.01, and ***p < 0.001 (compared with non-fasting). ^##^*p* < 0.01 and ^####^*p* < 0.0001 (compared with non-fasting controls). ^&&&&^*p* < 0.0001. PNX 5/6 partial nephrectomy, LP low-phosphate diet, SP standard-phosphate diet, HP high-phosphate diet. Mean ± SEM (**a**, **f**). Cosinor fit (**b**–**e**).

Plasma P exhibited significant circadian rhythmicity in control rats (ANOVA *p* < 0.0001), (Fig. 5). The rhythm was confirmed by cosinor analysis (*p* < 0.001), showing acrophase at 16:00 (Fig. 5b). In CKD rats, plasma P levels were higher in the PNX HP group (*p* < 0.01) and lower in the PNX LP group (*p* < 0.0001) as compared with controls (Fig. 5a). The significant CR of plasma P was present in CKD rats, but acrophase was shifted in all PNX groups (Fig. 5a, c–e). As such, the rhythm of PNX HP rats (cosinor analysis *p* < 0.0001) showed acrophase at 00:00 in contrast to the acrophase at 16:00 in controls. The PNX SP and LP groups showed shifted acrophase to 17:00 and 19:00, respectively, and both groups exhibited circadian rhythmicity (*p* < 0.05) confirmed by cosinor analysis (PNX LP; *p* < 0.05, PNX SP; *p* = 0.05).

**Fig. 6:**
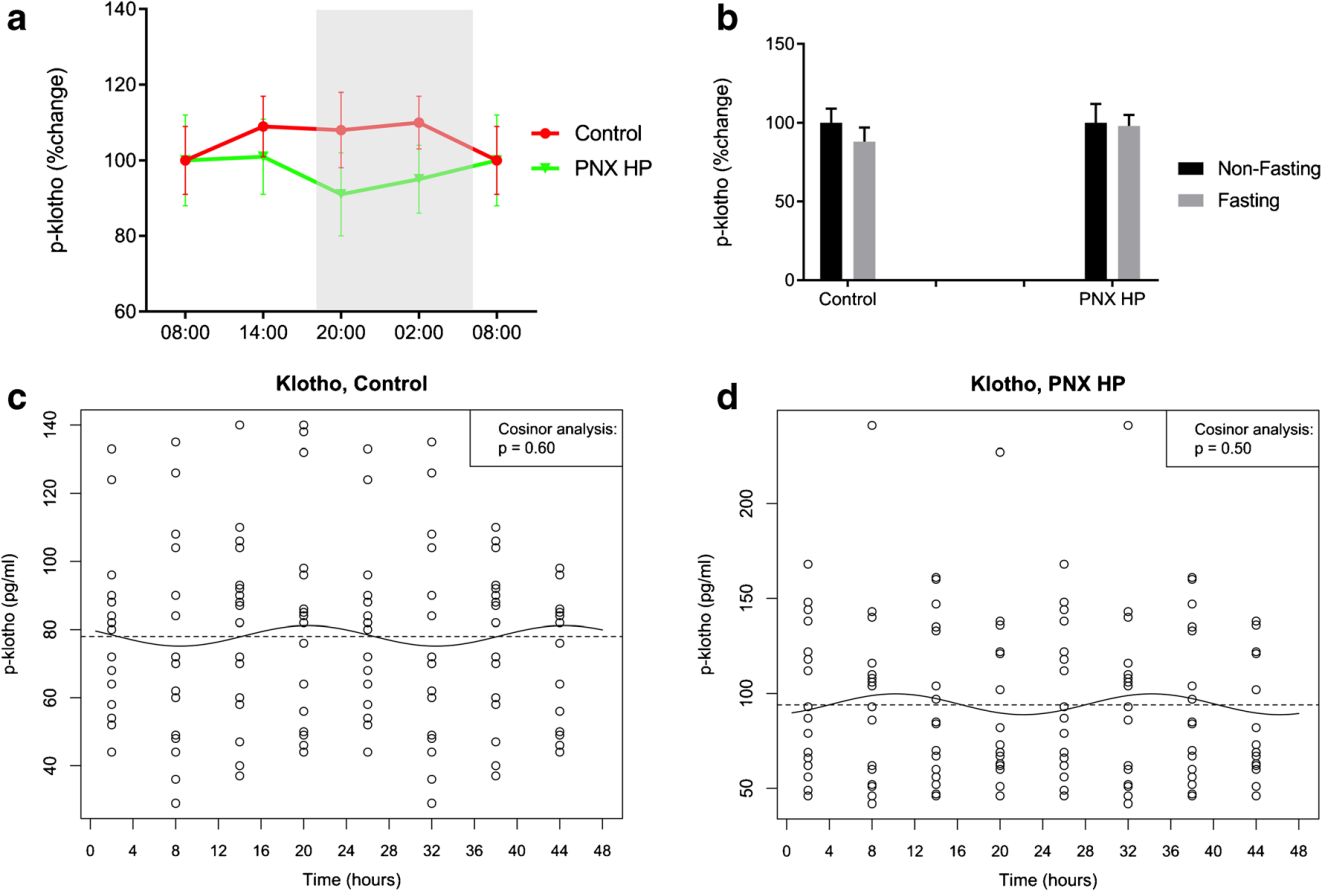
Plasma klotho does not exhibit circadian rhythm in control or CKD rats. **a** Stable levels of plasma klotho were found in both healthy controls (red) and PNX HP rats (green). **b** Non-fasting (black) and fasting (gray) plasma klotho levels in controls and PNX HP rats. Fasting did not affect plasma klotho in either group. **c**, **d** Circadian rhythm by cosinor analysis confirmed the lack of rhythmicity in controls (**c**) and PNX HP rats (**d**). PNX 5/6 partial nephrectomy, HP high-phosphate diet. Mean ± SEM (**a**, **b**). Cosinor fit (**b**, **d**).

Plasma klotho was measured only in controls and PNX HP rats. No difference was present between PNX HP and control rats and no CR was demonstrated (cosinor analysis *p* = 0.50 and *p* = 0.60), (Fig. 6a, c, d).

Plasma ionized and total calcium levels are presented in Tables 1 and 2. The cosinor analysis did not show circadian rhythmicity of these parameters (data not shown).

**Table 1.** Renal parameters and electrolytes

Plasma		Control				PNX LP			
		08:00	14:00	20:00	02:00	08:00	14:00	20:00	02:00
pH		7.37 ± 0.02	7.36 ± 0.02	7.34 ± 0.04	7.42 ± 0.01	7.30 ± 0.002	7.29 ± 0.02	7.37 ± 0.02	7.37 ± 0.02
K^+^	mM	5.8 ± 0.1	5.7 ± 0.2	5.6 ± 0.1	5.4 ± 0.1	6.2 ± 0.5	5.9 ± 0.4	6.3 ± 0.2	6.0 ± 0.2
Na^+^	mM	145 ± 1	146 ± 1	145 ± 1	145 ± 1	140 ± 1	142 ± 1	148 ± 2	140 ± 1
BUN	mM	7.2 ± 0.1	7.3 ± 0.2	7.7 ± 0.2	7.3 ± 0.1	21.2 ± 4.0	22.4 ± 5.0	21.3 ± 4.7	21.1 ± 4.2
tCa	mM	2.81 ± 0.02	2.78 ± 0.01	2.73 ± 0.04	2.77 ± 0.02	2.79 ± 0.05	2.84 ± 0.04	2.76 ± 0.04	2.81 ± 0.05
Ca^2+^	mM	1.43 ± 0.02	1.44 ± 0.02	1.39 ± 0.02	1.43 ± 0.02	1.42 ± 0.02	1.46 ± 0.02	1.51 ± 0.02	1.47 ± 0.02
Plasma		PNX SP				PNX HP			
		08:00	14:00	20:00	02:00	08:00	14:00	20:00	02:00
pH		7.31 ± 0.04	7.37 ± 0.02	7.35 ± 0.03	7.44 ± 0.01	7.39 ± 0.02	7.39 ± 0.01	7.28 ± 0.02	7.35 ± 0.04
K^+^	mM	6.4 ± 0.3	6.2 ± 0.3	6.5 ± 0.3	6.7 ± 0.3	6.4 ± 0.2	5.9 ± 0.2	6.7 ± 0.2	6.4 ± 0.2
Na^+^	mM	138 ± 1	142 ± 1	143 ± 1	147 ± 1	143 ± 1	143 ± 1	145 ± 1	145 ± 1
BUN	mM	15.6 ± 1.7	18.0 ± 2.7	17.8 ± 3.4	21.9 ± 4.4	13.2 ± 0.5	11.5 ± 0.6	12.6 ± 0.6	13.7 ± 0.6
tCa	mM	2.77 ± 0.04	2.79 ± 0.05	2.71 ± 0.05	2.77 ± 0.03	2.65 ± 0.04	2.64 ± 0.05	2.65 ± 0.07	2.65 ± 0.06
Ca^2+^	mM	1.36 ± 0.02	1.37 ± 0.02	1.38 ± 0.03	1.42 ± 0.02	1.30 ± 0.02	1.30 ± 0.02	1.28 ± 0.02	1.29 ± 0.03

Mean ± SEM.

*PNX* 5/6 partial nephrectomy, *LP* low-phosphate diet, *SP* standard-phosphate diet, *HP* high-phosphate diet

*K*^*+*^ potassium, *Na*^*+*^ sodium, *BUN* blood urea nitrogen, *tCa* total calcium, *Ca*^*2+*^ ionized calcium

**Table 2:** Non-fasting (NF) and fasting (F) renal parameters and electrolytes

Plasma		Control		PNX LP		PNX SP		PNX HP	
		NF	F	NF	F	NF	F	NF	F
pH	mM	7.37 ± 0.02	7.37 ± 0.02	7.27 ± 0.02	7.27 ± 0.03	7.39 ± 0.02	7.37 ± 0.02	7.41 ± 0.01	7.33 ± 0.01
K^+^	mM	5.6 ± 0.1	5.4 ± 0.2	6.5 ± 0.3	5.9 ± 0.1	6.6 ± 0.3	6.1 ± 0.2	6.4 ± 0.2	6.1 ± 0.2
Na^+^	mM	145 ± 1	144 ± 1	147 ± 1	142 ± 1	140 ± 0	142 ± 1	142 ± 1	142 ± 1
BUN	mM	7.0 ± 0.1	5.6 ± 0.1	25.5 ± 6.0	25.9 ± 6.3	16.4 ± 1.3	17.7 ± 1.6	12.6 ± 0.6	17.7 ± 1.8
tCa	mM	2.80 ± 0.02	2.75 ± 0.02	2.78 ± 0.06	2.87 ± 0.03	2.77 ± 0.05	2.84 ± 0.08	2.66 ± 0.04	2.55 ± 0.07
Ca^2+^	mM	1.43 ± 0.02	1.38 ± 0.01	1.43 ± 0.02	1.40 ± 0.01	1.33 ± 0.02	1.34 ± 0.02	1.29 ± 0.03	1.21 ± 0.03

Mean ± SEM.

*PNX* 5/6 partial nephrectomy, *LP* low-phosphate diet, *SP* standard-phosphate diet, *HP* high-phosphate diet

*K*^*+*^ potassium, *Na*^*+*^ sodium, *BUN* blood urea nitrogen, *tCa* total calcium, *Ca*^*2+*^ ionized calcium

Rhythm characteristics of cosinor analysis are presented in Supplementary Table 1.

### Effect of fasting on plasma activin A, FGF23, PTH, phosphate, and klotho

Plasma activin A increased by fasting in both normal (*p* < 0.05) and PNX LP rats (*p* < 0.01), but this response was obliterated in PNX SP and HP rats (Fig. 1f). All PNX groups had higher plasma activin A levels during non-fasting conditions as compared with controls (*p* < 0.01).

Fasting did not affect plasma FGF23 in control rats (Fig. 3f). In contrast, there was an increase in FGF23 in fasting CKD rats of 101% in PNX LP (*p* < 0.01), 111% in PNX SP (*p* < 0.001), and 74% in PNX HP rats (*p* < 0.05). PNX LP rats had lower non-fasting FGF23 levels compared with the control group (*p* < 0.0001) whereas both PNX SP and PNX HP had higher non-fasting FGF23 levels as compared with controls (*p* < 0.01 and *p* < 0.0001, respectively).

Fasting caused a large significant increase of 71% in plasma PTH in the control rats (*p* < 0.05), a 63% raise in PNX LP (ns), a 96% increase in PNX SP (*p* < 0.01), and a 53% raise in PNX HP (ns), (Fig. 4f).

Fasting resulted in increased levels of plasma P in all groups of experimental animals: 121% in PNX LP (*p* < 0.001), 48% in PNX SP (*p* < 0.001), and 22% in PNX HP (*p* < 0.05) as compared with a 5% increase in the control rats (*p* < 0.01), (Fig. 5f). Fasting did not affect plasma klotho in control rats nor in CKD rats (Fig. 6b).

#### Renal function and electrolytes

Renal parameters and electrolytes are presented in Tables 1 and 2. All PNX groups had higher azotemia and hyperkalemia as compared with control rats (*p* < 0.05).

## Discussion

Activin A is a new player in CKD-MBD of interest as a therapeutic target. The present investigation in the rat establishes for the first time the existence of a circadian rhythm (CR) of circulating activin A, which is disturbed in CKD rats and is associated with disturbed CRs of plasma parameters of CKD-MBD such as plasma phosphate (P) and the P-regulating hormones, PTH and FGF 23, indicating that disturbed circadian rhythmicity is a distinctive feature of CKD-MBD.

The present finding of a considerable circadian variation in the plasma activin A levels of more than 300% indicates a need to standardize sample collection protocols and time-specific reference intervals for the plasma levels. In the clinical setting, these diurnal variations of plasma activin A must be considered, when concentration changes of this CKD-MBD marker are interpreted.

The clinical importance of the present finding showing CR of circulating activin A has yet to be established, the same is the case for the significance of the disturbed rhythmicity and elevated levels of activin A. In humans, a disturbed CR is associated with increased risk of several diseases, and the risk of cardiovascular disease, metabolic syndrome, and cancer is increased in shift workers [25, 52, 71, 79].

Activin A is a member of the TGF-β superfamily. It is a cytokine expressed in a wide range of tissues and cells, where it regulates cellular differentiation, cell proliferation, apoptosis, and inflammation at an autocrine/paracrine level. Systemic activation of activin A receptors and increased circulating levels of activin A have been found in animal models of CKD-MBD [1, 58, 65, 72, 73, 80]. Recently, the first report was provided showing that systemic activin A level is elevated in humans with CKD, already at stage 2 [43]. Furthermore, systemic activin A levels are associated with aging and metabolic disorders where elevated activin A is an independent risk factor for prediabetes and diabetes [36]. In obese subjects, serum activin A levels correlate with parameters of the metabolic syndrome and left ventricular diastolic dysfunction [82]. Plasma activin A was found increased in patients with nonalcoholic fatty lever disease [61]. The potential disturbance of the CR of activin A in diabetes, metabolic syndrome, or in patients with CKD remains to be thoroughly examined.

A role of CR-related mechanisms in the pathogenesis of renal fibrosis has been proposed [10]. The key circadian gene, *Clock*, was shown to mediate the oscillation of TGF-β signaling and *Clock*-deficient mice had increased oxidative stress and renal fibrosis [10]. Concurrently, it was found that activation of the ALK pathway by TGF-β, activin, or variation of pH levels reset the circadian clock in Rat-1 fibroblast cells suggesting that ALK signaling is involved in activation of the peripheral circadian clocks [35].

The new concept of disruption in system biology in CKD as proposed by Hruska et al. [26] was based on the observation that the injured kidneys produce circulating signals which directly affect the vasculature, skeleton, and progression of renal fibrosis. Activin A is such a renal repair factor, which circulates in elevated levels in CKD. Inhibiting activin-signaling blocks vascular calcification, and renal fibrosis in CKD [1]. We have previously shown that inhibin β_A_ (*Inhba*), which codes for the β_A_ subunit (activin A is a homodimer composed of two inhibin β_A_ subunits), was not expressed in normal kidney, but was significantly induced in the injured kidney [58]. In the experimental model of unilateral ureter obstruction (UUO), we showed that activin A was induced in the obstructed kidney together with a twofold higher plasma level after 10 days of obstruction, while activin A was undetectable in the contralateral untouched kidney. Plasma levels did not increase in unilateral nephrectomized rats (UNX) and nor was *Inhba* detectable in the remnant UNX kidney. This indicates that kidney injury induces production of activin A with subsequent secretion to the circulation [58]. It suggests that activin A might be involved in the early pathophysiological changes occurring in CKD-MBD as recently supported by the group of Malluche et al. [43] and further indicates that injured kidney is an additional source of circulating activin A, which might contribute to disturbed circadian rhythmicity in CKD.

In an RNAseq analysis of calcified uremic rat aortas [65], we have previously found that the expression of the *Tgfbr1* gene, which codes for an alternative type 1 receptor downstream the activin type 2A receptor, was increased, and thus may contribute to the proposed importance of activin signaling in vascular calcification. As such, the renal expression of activin A in CKD may potentially change the physiological role of activin A in extra-renal tissues, including in the skeleton and vasculature.

Based on the present results, we hypothesize that the disturbed circadian rhythmicity of circulating activin A contributes to the disruption in system biology in CKD.

A clear impact of CKD and dietary P content was seen on the evaluated parameters. CKD induced a shift in the plasma P levels depending on the dietary P content, together with a disturbance in CR, in accordance with previous findings [5, 62].

The molecular circadian clock system is ubiquitously expressed throughout the body and drives CRs of numerous parameters and mechanisms, probably including the CR of plasma P. One explanation for the disturbed CR of P might be that CKD by itself affects the molecular circadian clock system and thereby alters the daily P fluctuations. Hypophosphatemia might have a regulating impact on the circadian clock genes, as recently shown in cardiac tissue [57]. However, PNX SP and LP rats exhibited similar circadian pattern, indicating that CKD rather than P content in the diet is the key modulator of the CR of plasma P in the present study.

It is still an open question whether a P sensor exists that regulates plasma P levels and potentially drives the CR of plasma P and P-regulating hormones, and it is also not known how the potential sensor might accommodate changes in time of day and CKD. P sensing in kidneys, bone, intestine, and parathyroids, which might regulate P homeostasis, could theoretically be involved [9, 56, 66]. A crystal model on the structure of the calcium-sensing receptor has recently revealed several P-binding sites and demonstrated that P reinforces the inactive state of the receptor [19]. As such, the calcium-sensing receptor can potentially be a part of a P-sensing mechanism in addition to others proposed in bone and intestine [6, 8, 39].

P in the diet is associated with increased plasma FGF23 [60] and recently a direct action through PiT-2 in bone has been described [8]. Whether P sensing in bone only relates to FGF23 or might be related to activin A secretion is currently unknown. A very recent study [54] indicated that the formation of daily oscillation of plasma P levels involves the Nampt/NAD+ system of the soft tissues, including the liver, intestine, and kidney.

In the present investigation, plasma activin A increased in fasting controls and PNX LP rats, but not in PNX SP or HP rats. The physiological cause of this finding is uncertain. Activin A is widely expressed and crucial during development. Its most acclaimed action is in reproductive physiology on the hypothalamic-pituitary-adrenal (HPA) axis [7]. It is believed that the HPA axis is activated during starvation [59]. As such, the increase in fasting controls could be the normal response of activin A to fasting, related to the HPA axis. Interestingly, the absent increase in plasma activin A occurs in fasting PNX SP and HP rats, who both have significant sHPT and elevated FGF23 (in contrast to controls and PNX LP rats). Thus, it could be speculated that the severely disturbed mineral homeostasis in these two groups might influence the natural response of activin A to fasting.

Fasting resulted in an increase of plasma P in all groups of rats. The P increase in fasting controls corroborates with the findings of previous investigations [15, 67], and the catabolic state of fasting has been shown to facilitate release of intracellular ions to the circulation (e.g., P), as known from studies on the refeeding syndrome [18]. Insulin administration causes a decrease in plasma P due to increased uptake of P in insulin-sensitive tissues [12]; hence, the hypoinsulinemia during fasting might also lead to leak of intracellular P and thereby to increased levels of plasma P.

In the present investigation, the discrepancy between the slight increase in plasma P in control rats and the massive increase in all PNX groups, underlines the impact of CKD on P homeostasis in the fasting condition. However, the preserved circadian variation of plasma P in all groups of CKD rats, independent of dietary P content and PTH levels, may point against intestinal P sensing and hormonal control of CR by PTH, which corroborates with a potential importance of the Nampt/NAD+ system [54]. The present data did not show circadian rhythmicity of plasma calcium or ionized calcium, which is in agreement with results of some previous studies [17].

The CR of plasma FGF23 in control rats and disturbed rhythm in CKD is a novel finding. Whether the secretion of FGF23 from osteocytes and osteoblasts is regulated by a local molecular circadian clock system or whether the CR of FGF23 is secondary to the CR of regulatory hormones (e.g., PTH) remains to be examined. In support of FGF23 being controlled by the some circadian rhythmicity, are data showing that bone mineralization exhibits daily fluctuations [53]. As such, induction of extra-skeletal FGF23 from bone marrow [75], kidney [48], and heart [14] has been shown in renal disease. Renal induction of FGF23 is located in the interstitium and not secreted [48], whereas FGF23 produced in the bone marrow seems to be secreted to the circulation through an erythropoietin-mediated mechanism [75]. Experimental myocardial infarction in rodents induces myocardial FGF23 and skeletal FGF23 together with a rise in circulating FGF23 [3] and the heart might therefore be capable of secreting FGF23. As such, like activin A, disturbed CR of FGF23 in CKD might not only be the consequence of abnormal bone metabolism, but also due to extra-skeletal secretion of the hormone.

## Conclusion

Activin A is a fascinating new factor in CKD-MBD of particular interest as a therapeutic target. The present study shows for the first time a circadian rhythm and a considerable circadian variation in plasma activin A levels. CKD resulted not only in an increased circulating level of activin A, but also in a disturbance in the circadian rhythm. A need to standardize sample collection protocols and reference intervals for the different plasma levels at different times of the day is stressed. Furthermore, CKD resulted in disturbed circadian rhythms of PTH, FGF23, and phosphate. As such, disturbed circadian rhythmicity in mineral homeostasis is an important feature of CKD-MBD.

## Electronic supplementary material

ESM 1 (PDF 81 kb)
