# Supplementary material for: Circadian rhythm of activin A and related parameters of mineral metabolism in normal and uremic rats
Source: Pflugers Arch. 2019 Jun 24;471(8):1079–94. doi: 10.1007/s00424-019-02291-2 (PMC6614158; doi:10.1007/s00424-019-02291-2)
Supplement: Supplementary file 1 — (PDF 81 kb) [file 424_2019_2291_MOESM1_ESM.pdf]

Supplementary Table 1. Cosinor analyses of circadian rhythms

	Significance	r^2	Acrophase	Mesor	Amplitude	Figure
**Activin A:**						
- Control	p<0.01	0.29	22:00	252pg/ml	134pg/ml	1b
- PNX LP	ns	-	-	-	-	1c
- PNX SP	ns	-	-	-	-	1d
- PNX HP	ns	-	-	-	-	1e
**FGF23:**						
- Control	p<0.05	0.06	13:00	799pg/ml	66pg/ml	3b
- PNX LP	ns	-	-	-	-	3c
- PNX SP	ns	-	-	-	-	3d
- PNX HP	p<0.05	0.07	09:00	3481pg/ml	568pg/ml	3e
**PTH:**						
- Control	p<0.0001	0.20	12:00	238pg/ml	147pg/ml	4b
- PNX LP	p<0.05	0.13	06:00	237pg/ml	165pg/ml	4c
- PNX SP	p<0.05	0.19	10:00	1885pg/ml	698pg/ml	4d
- PNX HP	ns	-	-	-	-	4e
**Phosphate:**						
- Control	p<0.0001	0.40	16:00	1.97mM	0.18mM	5b
- PNX LP	p<0.05	0.15	19:00	1.10mM	0.16mM	5c
- PNX SP	p=0.05	0.13	17:00	1.85mM	0.15mM	5d
- PNX HP	p<0.0001	0.13	00:00	2.70mM	0.25mM	5e
**Klotho:**						
- Control	ns	-	-	-	-	6c
- PNX HP	ns	-	-	-	-	6d

Cosinor analyses.
PNX: 5/6 partial nephrectomy. LP: Low phosphate diet. SP: Standard phosphate diet. HP: High phosphate diet.
Determination coefficient (r2), acrophase (rounded to nearest whole hour), Mesor (Midline Estimation Statistic of Rhythm), and amplitude are only presented in significant rhythms.

Circadian rhythm of activin A and related parameters of mineral metabolism in normal and uremic rats

Pflügers Archiv – European Journal of Physiology

Anders Nordholm MD, Søren Egstrand MD, Eva Gravesen MD, PhD, Maria L. Mace MD, PhD, Marya Morevati PhD, Klaus Olgaard MD, DMSc, Ewa Lewin, MD, DMSc*

*Correspondence to:
Ewa Lewin, MD, dr.med.,
ewa.lewin@regionh.dk

University of Copenhagen; Nephrological Department, Herlev Hospital, Herlev, Copenhagen, Denmark & Nephrological Department, Rigshospitalet, Copenhagen, Denmark
